# *Klebsiella pneumoniae*-induced multiple invasive abscesses: A case report and literature review

**DOI:** 10.1097/MD.0000000000017362

**Published:** 2019-09-27

**Authors:** Bin Wang, Peng Zhang, Yuxiang Li, Yang Wang

**Affiliations:** Department of Infectious Diseases, the First Hospital, Jilin University, Changchun, Jilin, China.

**Keywords:** abscess, antimicrobial susceptibility test, imaging examination, K1 serotype, *Klebsiella pneumoniae*

## Abstract

**Rationale:**

*Klebsiella pneumoniae* infection can induce multiple invasive abscesses, and the invasive infection is severe and life-threatening.

**Patient concerns:**

A 69-year-old previously healthy Chinese male presented with fever, chill, backache, and ocular pain.

**Diagnosis:**

The blood culture results indicated *Klebsiella pneumoniae* of the K1 serotype. Multiple invasive abscesses in liver, lung, eye, soft tissue, and central nervous system were identified by imaging examination. Subsequently, the patient experienced right ocular pain accompanied by visual disturbance. Tyndall sign was strongly positive, and lens opacity was observed by the ophthalmologist.

**Interventions:**

Full-dose and long-term treatment with meropenem was performed. Intraventricular injection of glass and anterior chamber puncture with antibiotics were performed twice. The patient also underwent an evacuation of the brain abscess.

**Outcomes:**

The patient's headache and lumbar backache were relieved, his ophthalmodynia disappeared, and his vision recovered after nearly 3 months of treatment.

**Lessons:**

Imaging examination is very important for severe *Klebsiella pneumoniae* infection. The choice of antibiotics is complex, and the antimicrobial regimen should be adjusted according to the assessment of illness and the therapeutic effect. Surgical intervention must be considered for patients with multiple invasive abscesses.

## 1 Introduction

*Klebsiella pneumoniae* (*K pneumoniae*) is a gram-negative, gas-producing, capsulated, nonmotile, enteric bacillus. It exists widely in nature and is one of the normal flora in the human intestine and oral cavity.^[1]^*K pneumoniae* has been implicated as a common cause of infection in the human body. There are a number of virulence factors that contribute to the pathogenicity of *K pneumoniae*, including a hypermucoviscosity-specific capsular serotype, especially K1 or K2, and the virulence genes *FimH*, *rmpA*, *uge*, *kfu*, and *alls*.^[2,3]^ K1 and K2 serotypes are more prevalent in invasive infections and are strongly associated with fatal outcomes. The invasive *K pneumoniae* strains were reported as having a hypermucoviscous phenotype associated with serotypes K1 and K2. Serotype K1 of *K pneumoniae* was associated with a hypermucoviscosity phenotype, was more resistant to neutrophil phagocytosis, and was reported as the major serotype that induced deep abscesses. ^[2–4]^ However, hypervirulent *K pneumoniae* strains and antibiotic-resistant *K pneumoniae* have emerged separately across the world. *K pneumoniae* is one of several bacteria that have exhibited a dramatic increase in antibiotic resistance in the past decades. Perhaps because of the selective pressure of treating extended-spectrum β-lactamase infections with carbapenems, carbapenem resistance has emerged, and *K pneumoniae* is the most common carbapenem-resistant *Enterobacteriaceae*.^[5,6]^ As a result, treatment has become more challenging. There is an increasing mortality and morbidity associated with this organism.^[7]^ In China, the isolation rate of *K pneumoniae* increased from 2.4% to 13.4% year by year, and carbapenem resistance among *K pneumoniae* isolates increased from 2.9% to 13.4%.^[8]^

Herein, we present an adult patient with multiple invasive abscesses induced by *K pneumoniae* with definite K1 serotyping. He was cured after long-term antibiotic treatment and surgical treatment.

## 2 Case representation

The patient was a 69-year-old previously healthy Chinese male with no significant medical history and surgical history but with a history of lumbar intervertebral disc protrusion for >10 years. He presented with fever, chills, and backache for 10 days and had suddenly experienced fever up to 40°C. He sometimes experienced ocular pain without visual disturbance. The patient was treated with moxifloxacin for 3 days (400 mg, intravenous drip [i.v. D]) and merocillin/sulbactam sodium (3.75, q8 h, i.v. D) for 2 days, and then his body temperature fluctuated from 37°C to 38.8°C.

**Figure 1:**
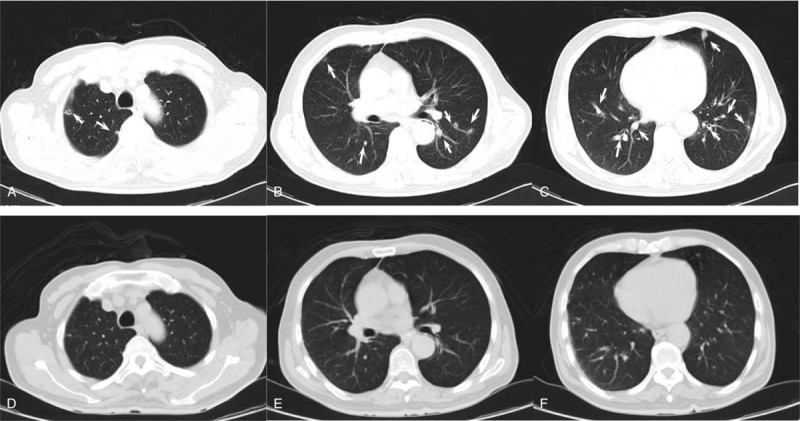
CT scan of the lung. (A, B and C) Initial CT lung images after hospitalization in our hospital. Multiple nodules and some with a small cavity were observed (marked with white arrows). (D, E and F) The follow-up CT lung images from 2 months later.

**Figure 2:**
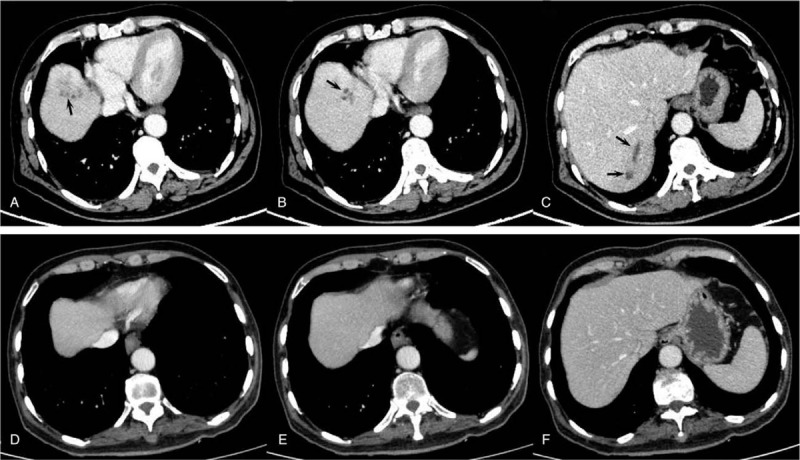
The contrast-enhanced CT scan of the abdomen. (A, B and C) The initial contrast-enhanced CT abdomen images after hospitalization in our hospital. Multiple round mild hypodense areas were observed and are marked with white arrows. (D, E and F) The follow-up CT abdomen images from two months later.

**Figure 3:**
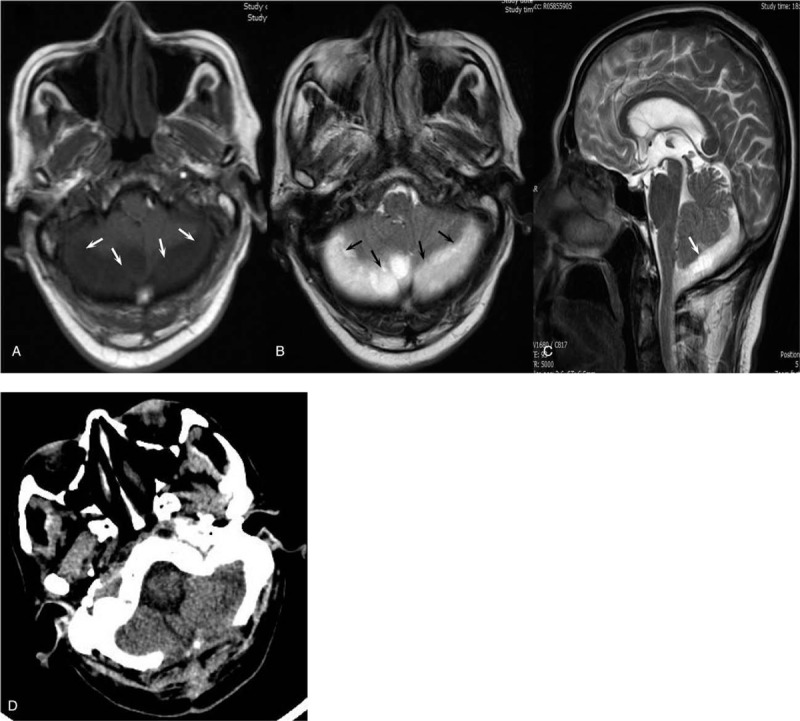
Imaging examination of the brain. (A, B and C) The initial contrast-enhanced MRI brain images. (A) T1 image. (B) T2 image. (C) T2 images of the sagittal position. The bilateral posterior fossa subdural effusion, subsequent hydrocephalus, and edema in the ventricle are displayed and marked with white arrows. (D) The follow-up CT of the brain after excision of the brain abscess.

**Figure 4:**
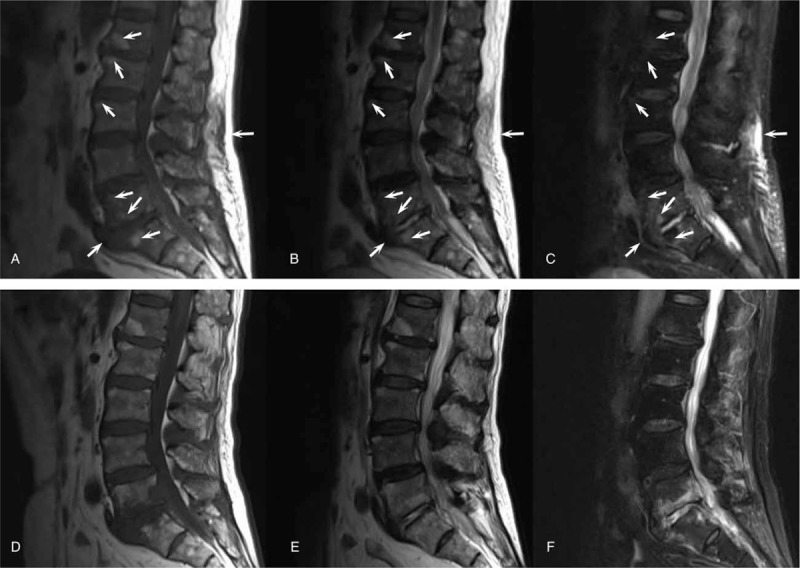
MRI of the lumbar region. (A, B and C) The initial contrast-enhanced MRI lumbar images. (A) T1 image. (B) T2 image. (C) Fat-suppressed image. Abnormal signal shadows in lumbar vertebrae 1-5 and sacrum 1, thickening of the soft tissue around lumbar vertebra 5 and sacrum 1, and an abnormal signal shadow from the soft tissue of the lower back were observed (marked with a white arrow). (D, E, and F) Follow-up MRI images 2 months later. (A) T1 image. (B) T2 image. (C) Fat-suppressed image.

Blood analysis revealed signs of inflammation: C-reactive protein level of 316 mg/L and procalcitonin level of 10.88 ng/mL. WBC (171,000 cells/mL) and the percentage of neutrophils (92%) increased, and thrombocytes were in the reference range. Liver enzymes were increased (aspartate aminotransferase 56.2 U/L, alanine aminotransferase 67.6 U/L and gamma glutamyl transferase 179.9 U/L). To exclude special bacterial infections, serological examination of *Brucella* and interferon γ release assays for tuberculosis were tested, and the results were negative. Kidney function was at the normal level. An initial lung computed tomography (CT) scan showed that multiple nodules with small cavities were observed in the lungs (Fig. 1A–C). An initial abdominal CT scan showed that there were several round, mild, hypodense areas; the boundary was not clear, and the edges were slightly enhanced (Fig. 2A–C). The results of a CT scan indicated that the patient had a hematogenic pulmonary abscess and liver abscess. *K pneumoniae* was isolated from blood and was found to be sensitive to several antibiotics (including gentamicin, aztreonam, ceftriaxone, levofloxacin, piperacillin-tazobactam, cefepime, imipenem, and meropenem); the isolate was identified as a K1 serotype based on the positive findings of *magA* gene detected by polymerase chain reaction (PCR). Brain magnetic resonance imaging (MRI) revealed a bilateral posterior fossa subdural effusion, subsequent hydrocephalus, and edema in the ventricle (Fig. 3A–C). Because of obvious lumbar backache, MRI of the lumbar region was required for inspection. The results showed abnormal signal shadows in lumbar vertebrae 1–5 and sacrum 1, thickening soft tissue around lumbar vertebra 5 and sacrum 1, and an abnormal signal shadow in the soft tissue of the lower back (Fig. 4A–C) (Table 1).

**Table 1:**
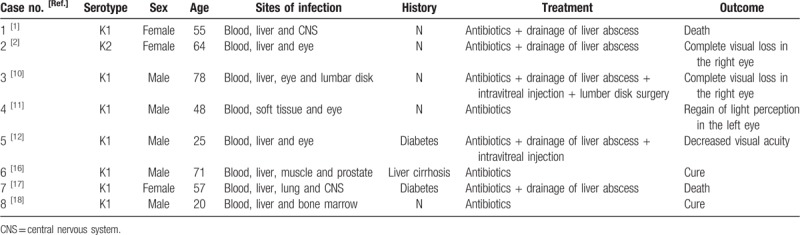
Summary of *Klebsiella pneumoniae*-induced multiple invasive abscess cases reported in the literature.

Treatment was started with cefepime (2.0, q8 h, i.v. D, for 5 days) and then was changed to meropenem (2.0, q8 h, i.v. D) after verifying central nervous system involvement. After treatment with meropenem for 2 days, the body temperature of the patient recovered to the normal level, but his backache was not markedly relieved. Next, a CT scan of the lumbar vertebrae was performed, and the results showed that a high-density shadow was located in the lumbar 5 and sacral 1 vertebral bodies, and the surrounding soft tissue was thickened, especially around the left psoas (Fig. 4A–C). After 15 days of treatment, the patient experienced right ocular pain aggravated with visual disturbance. Tyndall sign was strongly positive, and lens opacity was observed by an ophthalmologist. Intraventricular injection of glass and an anterior chamber puncture with ceftazidime (0.4 mg) and amikacin (0.4 mg) were performed twice. The patient's ophthalmodynia disappeared, and his vision recovered. The rescrutinized CT scan of the lung and liver showed that the focus of the abscesses was decreased greatly and even disappeared (Fig. 1D–F and Fig. 2D–F). Excision of brain abscess was performed to remove the brain abscess after 23 days of antibiotic treatment because of a refractory headache and the unreduced volume of the brain abscess. After the surgery, the patient's headache and backache were relieved, and his stiff neck disappeared. The results of the CT scan of the brain showed recovery (Fig. 3D). The lumbar MRI was reexamined, and the results showed that the abnormal signal shadow in the soft tissue of the lower back disappeared (Fig. 4D–F). After <3 months of treatment, the patient left the hospital without any discomfort.

## 3 Discussion

*K pneumoniae* is a frequently isolated, well-established bacterial pathogen.^[9]^ A *K pneumoniae* infection can involve the liver, lung, urinary tract, abdominal cavity, blood, and central nervous system, and it can be life-threatening.^[6]^ Here, we report a case of *K pneumoniae*-induced multiple invasive abscesses exhibiting a K1 serotype by detection of the *magA* gene via PCR assay. The K1 serotype of *K pneumoniae* is the major serotype that induces deep abscesses.^[4,11,12]^ Invasive infection caused by *K pneumoniae* is common in immunosuppressed patients, but it could also be observed in some immunocompetent patients. It was reported that prevalence of *K pneumoniae* in healthy adults was 75%, with a high prevalence (23%) of serotype K1 or K2 isolates in Taiwan.^[13]^ The high prevalence of virulent *K pneumoniae* strains in patients of Asian descent is probably why the prevalence of this invasive syndrome is so high in this population. In this case, K1 serotype is the major risk factor. Although the bacteria were identified as sensitive to most kinds of antibiotics, multiple invasive abscesses in this case were still life-threatening. A more hypervirulent gene, such as *rmpA*, should be detected by PCR.^[14]^ However, the samples of the isolated bacteria were not preserved, and unfortunately, no additional DNA could be used for further examination.^[3,14]^

Most patients with *K pneumoniae* invasive infection have a severe infection, and some of them die from this life-threatening condition, especially those with bloodstream infection and multiple invasive abscesses.^[15]^ The most common manifestations of metastatic infection are endophthalmitis, meningitis, and brain abscesses. Other manifestations include lumbar or cervical spondylitis and discitis, septic pulmonary emboli, lung abscesses, splenic abscesses, necrotizing fasciitis, neck abscesses, cerebral abscesses, purulent meningitis, otitis media, osteomyelitis, arthritis, prostate abscesses, pylephlebitis, and psoas muscle abscesses.^[10–12,16–18]^ The outcome of some patients with *K pneumoniae*-induced endophthalmitis could be complete visual loss.^[2,10–12]^ Furthermore, severe *K pneumoniae* infection could result in death because of intracranial hypertension, cerebral hernia, and sepsis.^[1,17]^ CT and enhanced CT scans are the best imaging examinations for discovering deep abscesses, especially those in the lungs, brain, pelvis, spine, and abdomen. MRI and enhanced MRI could also be used to scan for deep focal infection in the brain, spine, and abdomen. A specialist is also needed when specific infected organs are involved. In this case, the *K pneumoniae*-induced infection involved the liver, lung, blood, eye, soft tissue, and central nervous system. The patient's condition deteriorated rapidly. The multiple abscesses in the lung and liver were very small, and drainage could not be performed. Fortunately, the patient was examined and treated by an ophthalmologist after he felt ocular pain aggravated with visual disturbance. Excision of brain abscess was performed to remove the brain abscess. His outcome could have been life-threatening without these surgical treatments combined with appropriate antibiotic therapy.

Moxifloxacin, merocillin/sulbactam sodium, and cefepime were used in this case. However, the patient's clinical symptoms gradually worsened, although *K pneumoniae* organisms isolated from the blood were identified as being sensitive to these antibiotics. However, his body temperature and other clinical manifestations improved after treatment with meropenem. In this case, the patient had a severe case with a bloodstream infection and multiple invasive abscesses. The susceptibility of bacteria to antibiotics *in vivo* is not always the same as the results *in vitro*,^[19]^ but can be influenced by many factors, such as host immunology and biofilm formation.^[20]^ Susceptibility test results are important and predictive, but antibiotic behaviors must be evaluated *in vitro* and *in vivo* to confirm their suitability for therapeutic use.^[21]^ The choice of antibiotics needs to change according to the antimicrobial susceptibility test results *in vitro*, assessment of the illness, and therapeutic effect of the antibiotics *in vivo*. Surgical intervention must be evaluated for patients with multiple invasive abscesses.

This case might contribute to improving our understanding of the importance of imaging examinations of severe *K pneumoniae* infection.

## 4 Patient consent

Written informed consent was obtained from the patient for publication of clinical data, including all images in this case report.

## Acknowledgments

The authors thank radiological technicians and neurosurgeons for their efforts in clinical diagnosis and management of this patient.

## Author contributions

**Conceptualization:** Yang Wang.

**Data curation:** Bin Wang, Peng Zhang.

**Formal analysis:** Peng Zhang.

**Funding acquisition:** Yang Wang.

**Investigation:** Bin Wang, Peng Zhang, Yuxiang Li.

**Methodology:** Bin Wang, Yuxiang Li.

**Project administration:** Peng Zhang, Yuxiang Li.

**Software:** Bin Wang.

**Supervision:** Yuxiang Li.

**Validation:** Yang Wang.

**Visualization:** Peng Zhang, Yuxiang Li.

**Writing – original draft:** Bin Wang, Yang Wang.

**Writing – review & editing:** Yang Wang.

## References

[1] Qian Y Wong CC Lai SC *Klebsiella pneumoniae* invasive liver abscess syndrome with purulent meningitis and septic shock: a case from mainland China. World J Gastroenterol 2016;22:2861–6. 26973425 10.3748/wjg.v22.i9.2861 PMC4778009

[2] Seo R Kudo D Gu Y Invasive liver abscess syndrome caused by Klebsiella pneumoniae with definite K2 serotyping in Japan: a case report. Surg Case Rep 2016;2:72. 27457077 10.1186/s40792-016-0201-2 PMC4960081

[3] Remya P Shanthi M Sekar U Occurrence and characterization of hyperviscous K1 and K2 serotype in Klebsiella pneumoniae. J Lab Physicians 2018;10:283–8. 30078963 10.4103/JLP.JLP_48_18 PMC6052812

[4] Qu TT Zhou JC Jiang Y Clinical and microbiological characteristics of Klebsiella pneumoniae liver abscess in East China. BMC Infect Dis 2015;15:161. 25886859 10.1186/s12879-015-0899-7 PMC4381403

[5] Chiang TT Yang YS Yeh KM Quantification and comparison of virulence and characteristics of different variants of carbapenemase-producing Klebsiella pneumoniae clinical isolates from Taiwan and the United States. J Microbiol Immunol Infect 2016;49:83–90. 26514941 10.1016/j.jmii.2015.08.011

[6] Martin RM Bachman MA Colonization, infection, and the accessory genome of Klebsiella pneumoniae. Front Cell Infect Microbiol 2018;8:4. 29404282 10.3389/fcimb.2018.00004 PMC5786545

[7] Chew KL Lin RTP Teo JWP Klebsiella pneumoniae in Singapore: hypervirulent infections and the carbapenemase threat. Front Cell Infect Microbiol 2017;7:515. 29312894 10.3389/fcimb.2017.00515 PMC5732907

[8] Hu FP Guo Y Zhu DM Resistance trends among clinical isolates in China reported from CHINET surveillance of bacterial resistance, 2005–2014. Resistance trends among clinical isolates in China reported from CHINET surveillance of bacterial resistance, 2005–2014. Clin Microbiol Infect 2016;22 suppl 1:S9–14. 27000156 10.1016/j.cmi.2016.01.001

[9] Guo Y Wang S Zhan L Microbiological and clinical characteristics of hypermucoviscous Klebsiella pneumoniae isolates associated with invasive infections in China. Front Cell Infect Microbiol 2017;7:24. 28203549 10.3389/fcimb.2017.00024 PMC5286779

[10] Baekby M Hegedüs N Sandahl TD Hypervirulent Klebsiella pneumoniae K1 liver abscess and endogenous endophthalmitis in a Caucasian man. Clin Case Rep 2018;6:1618–23. 30147917 10.1002/ccr3.1696 PMC6099053

[11] Chiu HHC Francisco CN Bruno R Hypermucoviscous capsular 1 (K1) serotype Klebsiella pneumoniae necrotising fasciitis and metastatic endophthalmitis. BMJ Case Rep 2018;11:pii:e226096. 10.1136/bcr-2018-226096 PMC6301438 30567095

[12] Xu M Li A Kong H Endogenous endophthalmitis caused by a multidrug-resistant hypervirulent Klebsiella pneumoniae strain belonging to a novel single locus variant of ST23: first case report in China. BMC Infect Dis 2018;18:669. 30558549 10.1186/s12879-018-3543-5 PMC6296127

[13] Lin YT Siu LK Lin JC Seroepidemiology of Klebsiella pneumoniae colonizing the intestinal tract of healthy Chinese and overseas Chinese adults in Asian countries. BMC Microbiol 2012;12:13. 22260182 10.1186/1471-2180-12-13 PMC3273430

[14] Yeh KM Kurup A Siu LK Capsular serotype K1 or K2, rather than magA and rmpA, is a major virulence determinant for Klebsiella pneumoniae liver abscess in Singapore and Taiwan. J Clin Microbiol 2007;45:466–71. 17151209 10.1128/JCM.01150-06 PMC1829066

[15] Xu M Fu Y Kong H Bloodstream infections caused by Klebsiella pneumoniae: prevalence of blaKPC, virulence factors and their impacts on clinical outcome. BMC Infect Dis 2018;18:358. 30064360 10.1186/s12879-018-3263-x PMC6069789

[16] Liao CY Yang YS Yeh YC Invasive liver abscess syndrome predisposed by Klebsiella pneumoniae related prostate abscess in a nondiabetic patient: a case report. BMC Res Notes 2016;9:395. 27506523 10.1186/s13104-016-2188-y PMC4977897

[17] Coutinho RL Visconde MF Descio FJ Community-acquired invasive liver abscess syndrome caused by a K1 serotype Klebsiella pneumoniae isolate in Brazil: a case report of hypervirulent ST23. Mem Inst Oswaldo Cruz 2014;109:970–1. 25411006 10.1590/0074-0276140196 PMC4296507

[18] Sturm E Tai A Lin B Bilateral osteomyelitis and liver abscess caused by hypervirulent Klebsiella pneumoniae- a rare clinical manifestation (case report). BMC Infect Dis 2018;18:380. 30086713 10.1186/s12879-018-3277-4 PMC6081821

[19] Lalitha P Srinivasan M Manikandan P Relationship of in vitro susceptibility to moxifloxacin and in vivo clinical outcome in bacterial keratitis. Clin Infect Dis 2012;54:1381–7. 22447793 10.1093/cid/cis189 PMC3334362

[20] He S He H Chen Y In vitro and in vivo analysis of antimicrobial agents alone and in combination against multi-drug resistant Acinetobacter baumannii. Front Microbiol 2015;6:507. 26074898 10.3389/fmicb.2015.00507 PMC4444844

[21] Akhi MT Asghari B Nahaei MR Comparison of in vitro activities of meropenem productions on Klebsiella pneumoniae isolated from hospitalized patients. GMS Hyg Infect Control 2014;9:Doc12. 25152857 10.3205/dgkh000232 PMC4141630

